# Pathological findings and morphologic correlation of the lungs of autopsied patients with SARS-CoV-2 infection in the Brazilian Amazon using transmission electron microscopy

**DOI:** 10.1590/0037-8682-0850-2020

**Published:** 2021-04-12

**Authors:** Monique Freire Santana, Rebecca Augusta de Araújo Pinto, Bruna Hilzendeger Marcon, Lia Carolina Almeida Soares de Medeiros, Thiago Barros do Nascimento de Morais, Lucas Castanhola Dias, Lorenna Pereira de Souza, Gisely Cardoso de Melo, Wuelton Marcelo Monteiro, Marcus Vinicius Guimarães Lacerda, Fernando Almeida Val, Pritesh Jaychand Lalwani, Luiz Carlos de Lima Ferreira

**Affiliations:** 1 Fundação Centro de Controle de Oncologia do Estado do Amazonas, Departamento de Ensino e Pesquisa, Manaus, AM, Brasil.; 2 Universidade do Estado do Amazonas, Programa de Pós-Graduação em Medicina Tropical, Manaus, AM, Brasil.; 3 Fundação de Medicina Tropical Dr. Heitor Vieira Dourado, Manaus, AM, Brasil.; 4 Hospital Universitário Getúlio Vargas, Programa de Residência Médica em Patologia, Manaus, AM, Brasil.; 5 Fundação Oswaldo Cruz, Instituto Carlos Chagas, Curitiba, PR, Brasil.; 6 Fundação Oswaldo Cruz, Instituto de Pesquisas Leônidas & Maria Deane, Manaus, AM, Brasil.; 7 Instituto Nacional de Pesquisas da Amazônia, Laboratório Temático de Microscopia e Nanotecnologia, Manaus, AM, Brasil.; 8 Universidade Federal do Amazonas, Programa de Pós-Graduação em Ciências da Saúde, Manaus, AM, Brasil.

**Keywords:** COVID-19, Severe acute respiratory syndrome 2, Lung pathology, Electron microscopy, Diffuse alveolar damage, Postmortem evidence

## Abstract

**INTRODUCTION:**

Electron microscopy (EM) is a rapid and effective tool that can be used to create images of a whole spectrum of virus-host interactions and, as such, has long been used in the discovery and description of viral mechanisms.

**METHODS:**

Electron microscopy was used to evaluate the pulmonary pathologies of postmortem lung sections from three patients who died from infection with SARS-associated coronavirus 2 (SARS-CoV-2), a new member of the *Coronaviridae* family.

**RESULTS:**

Diffuse alveolar damage (DAD) was predominant in all three patients. The early exudative stage was characterized principally by edema and extravasation of red blood cells into the alveolar space with injury to the alveolar epithelial cells; this was followed by detachment, apoptosis, and necrosis of type I and II pneumocytes. The capillaries exhibited congestion, exposure of the basement membrane from denuded endothelial cells, platelet adhesion, fibrin thrombi, and rupture of the capillary walls. The proliferative stage was characterized by pronounced proliferation of type II alveolar pneumocytes and multinucleated giant cells. The cytopathic effect of SARS-CoV-2 was observed both in degenerated type II pneumocytes and freely circulating in the alveoli, with components from virions, macrophages, lymphocytes, and cellular debris.

**CONCLUSIONS:**

Viral particles consistent with the characteristics of SARS-CoV-2 were observed mainly in degenerated pneumocytes, in the endothelium, or freely circulating in the alveoli. In the final stage of illness, the alveolar spaces were replaced by fibrosis.

## INTRODUCTION

Just before the beginning of 2020, the world was astonished by the outbreak of a new type of pneumonia from Wuhan, China. The culprit, a novel beta-coronavirus, was eventually identified and named SARS-CoV-2 (severe acute respiratory coronavirus 2), and the name coronavirus disease 19 (COVID-19) was given to the illness it caused in humans. This RNA virus likely originated from pangolins and bats^1^ and is associated with a broad spectrum of clinical manifestations; as such, cases can vary in presentation from asymptomatic infection to severe illness. Coronaviruses possess a positive-sense single-strand RNA genome which is responsible for encoding structural and non-structural proteins. The structural protein group includes the nucleocapsid protein, membrane glycoprotein, small envelope protein, and spike glycoprotein^2^. 

Electron microscopy (EM) is a rapid and effective tool that can be used to create images of a whole spectrum of virus-host interactions and, as such, has long been used in the discovery and description of viral mechanisms^3^. One of the main advantages of EM is its ability to recognize pathogenic agents without relying on organism-specific reagents. Even in the age of molecular diagnostics, EM remains important in the quality control of molecular techniques and helps detect new and unusual outbreaks^4^. 

Electron microscopy can be used to analyze SARS-CoV-2 and elucidate the physiopathologic mechanisms behind pulmonary involvement in COVID-19. Currently, there are few studies that described and correlated the ultrastructural findings in SARS-CoV-2 infection with histopathologic lesions. This paper describes the endothelial and pulmonary epithelial lesions caused by COVID-19 using transmission electron microscopy (TEM) on samples obtained from patients during necropsy. 

## METHODS

Three patients who were infected with SARS-CoV-2, as confirmed by reverse transcription-polymerase chain reaction and compatible clinical and radiological findings, were submitted for autopsy. Lung samples were collected using minimally invasive tissue sampling (MITS, previously called minimally invasive autopsy) until six hours after death using techniques described in previous literature^5^. The lungs were randomly punctured using a 14-gauge needle for pathological analysis with one sample collected from each lung. Immediately after MITS, a complete autopsy was conducted by a pathologist and an autopsy technician to obtain larger samples. The cases described in this paper were randomly selected from autopsies of fifty-five patients with SARS-CoV-2 infection. 

The collected tissue samples were fixed with a solution of 2.5% glutaraldehyde and 4% paraformaldehyde in 100 mM sodium cacodylate buffer and stored at 4 °C for further processing. The samples were then washed in 100 mM sodium cacodylate buffer (3 times for 10 min each at pH 7.2) . For sample P1 (patient 1), the tissue fragments were post-fixed with 1% osmium tetroxide in the same buffer for 2 hours, washed, and subjected to block contrast using 5% aqueous uranyl acetate for 12 hours at 4 °C. The tissue samples from P1 and P3 were post-fixed with 1% osmium tetroxide, 0.8% potassium ferricyanide, and 5 mM calcium chloride in 0.1 M sodium cacodylate buffer for 1 hour and 20 minutes followed by washing. The samples were then dehydrated using a graded series of ethanol (P1) or acetone (P1 and P3). For P1, the tissue fragments were treated with propylene oxide twice for 15 minutes each before being embedded in Durcupan-ACM Fluka^©^ resin. The samples from P2 and P3 were embedded in 812 resin (Electron Microscopy Sciences, EMS). 

Semi-thin sections (500 nm on average) were obtained using a Leica EM UC6 ultramicrotome and collected on glass slides. The sections were then successively stained using 0.5% methylene blue, 0.1% fuchsine, and 5% ethanol aqueous solutions before being analyzed using a Nikon 80i microscope. 

Ultrathin sections (70 nm on average) were obtained using a Leica EM UC6 ultramicrotome and collected on copper grids. The samples were contrast-stained with 5% uranyl acetate for 50 min and lead citrate for 5 min. Analysis was then performed using a Jeol JEM1400-Plus transmission electron microscope. 

## RESULTS

**FIGURE 1:**
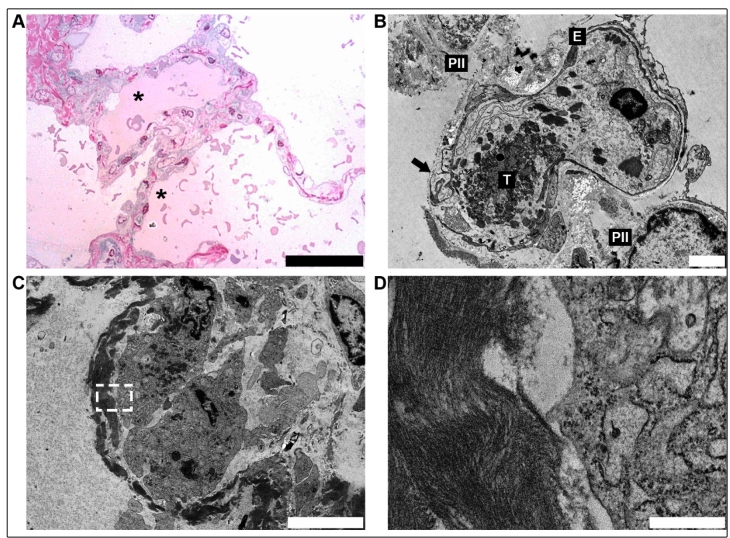
**(A)** Semi-thin section of the lung with an early exudative stage that was characterized principally by intra-alveolar edema (*, superior) and erythrocyte leakage (*, inferior), as well as injury to the alveolar epithelial cells (scale bar = 50 µm). **(B)** Electron microscopy of the pulmonary capillary showing disruption of the epithelial-endothelial barrier. Fibrin thrombi (T) inside the capillary. Type II pneumocytes (PII), endothelial cell (E) (scale bar = 2 µm). **(C)** Cellular debris, with nuclei in pyknosis and karyolysis of a type II pneumocyte filled by amorphous material, featuring a hyaline membrane (white, serrated edge rectangle) (scale bar = 5 µm). **(D)** Higher magnification of the dashed area in image C showing the fibrilar aspect of the material lining the alveoli (scale bar = 0.5 µm).

**FIGURE 2:**
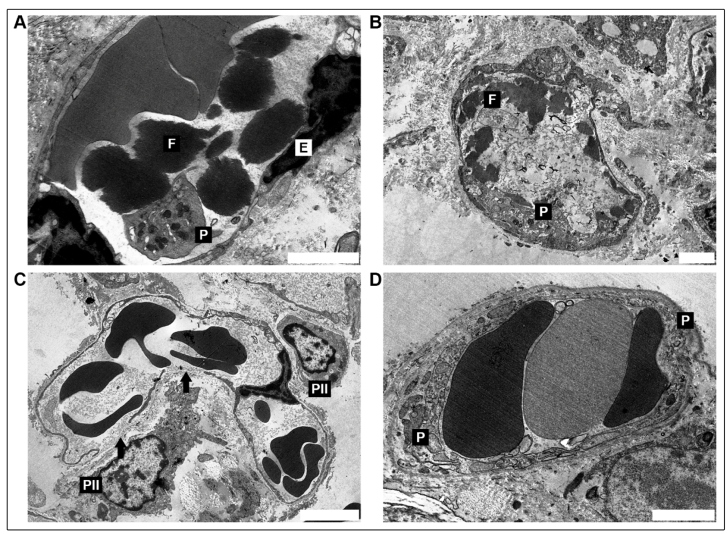
**(A)** Endothelial cell (right, bottom) with enlargement of nuclei and pyknotic chromatin (E). Inside the capillary, fibrin (F) platelet adhesion (P) (P3, scale bar = 2 µm). **(B)** Capillary dilatation with degeneration of endothelial cells exposing the basal membrane with platelet adhesion (P) and fibrin (F) formation (P1, scale bar = 2 µm). **(C)** Capillary dilatation, with disruption of the basement membrane and detachment (arrow) of alveolar epithelial cells (PII) in two points; type II pneumocyte necrosis (P1, scale bar = 5 µm). **(D)** Thinning of the basement membrane of the capillary endothelium, with rupture and platelet aggregation with attachment to the endothelium (P3, scale bar = 2 µm).

All three patients were male and aged 34 (P1), 81 (P2), and 66 (P3) years old. All three had sepsis and severe acute respiratory failure, and they all underwent orotracheal intubation. Patients P1, P2, and P3 were hospitalized for 12, 13, and 2 days, respectively, and the time between symptom onset and death was 17, 21, and 12 days, respectively. During hospitalization, P1 and P3 were medicated with anticoagulants, antibiotics, and oseltamivir, while P2 received the same drugs as P1 and P3 but with the addition of corticosteroids. Regarding laboratory data, P2 was the only patient who presented with a hemoglobin count below the reference value at 10.6 g/dL (RV 11.0 - 16.0 g/dL), while P1 and P3 remained within normal laboratory values (13.0 and 11.0 g/dL, respectively). P3 had thrombocytosis with a platelet count of 437 (x 109/L) (RV 100 - 300 x 109/L). P1 and P2 had normal platelet values at 184 and 227 (x 109/L), respectively. In addition to thrombocytosis, P3 also had leukocytosis with a count of 16.22 (x 109/L). P1 and P2 had leukocyte counts of 10.88 and 6.59 (x 109/L), respectively (RV 4.00 - 10.00 x 109/L). Post-mortem examination of the samples in hematoxyline and eosin sections showed advanced diffuse alveolar damage (DAD). The early exudative stage was characterized principally by edema and the presence of red blood cells in the alveolar space (Figure 1A). Other findings in this stage include injured alveolar epithelial cells (characterized by detachment, apoptosis, and necrosis of type I and II pneumocytes) and the presence of edema, red blood cells, and necrotic cell debris in the airspaces. The changes in the capillary wall were varied and characterized by congestion, exposure of the basement membrane due to denuded endothelial cells, platelet adhesion, formation of fibrin thrombi (Figure 1B), rupture of the capillary wall with an overflow of red blood cells (Figure 1A), and the presence of plasma-derived proteinaceous material which contributed to the edema. Hyaline membranes are the hallmark sign of the exudative phase of DAD and are described as the presence of amorphous material with a granular to fibrillar matrix located amidst occasional disintegrating and necrotic cell organelles (Figure 1C and D). Other endothelial alterations are shown in Figure 2.

**FIGURE 3:**
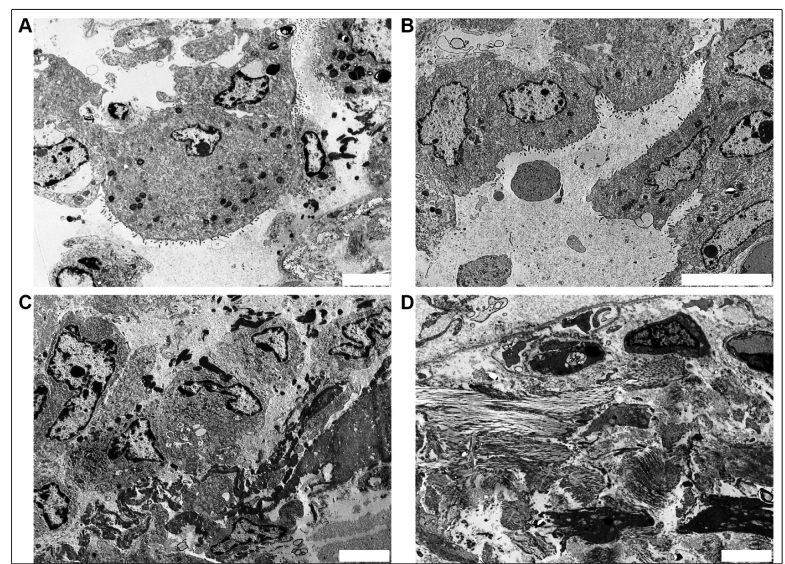
**(A)** Electron microscopy of the lung showing hyperplastic type II pneumocytes displaying striking cytological atypia, including cytomegaly in patient 3 with considerable enlargement of the cytoplasm and lysosomes (scale bar = 5 µm). **(B)** Electron microscopy showing the exsudative stage of diffuse alveolar damage with hyperplasia of type II pneumocytes forming syncytial cells (scale bar = 10 µm). **(C)** Cellular debris and fibrin in the alveolar space (scale bar = 5 µm). **(D)** Fibroblast proliferation associated with deposition of collagen and reticulin fibers, as well as obliteration of alveolar spaces (scale bar = 5 µm).

The lungs of P1 and P3 showed plugs of organizing fibroblastic spaces within the alveolar spaces and interstitium​, while bacterial bronchopneumonia was observed bilaterally at the base in P3. P2 showed type II pneumocyte desquamation, lymphocytic interstitial inflammation, and squamous metaplasia. The findings in all patients suggest the presence of diffuse alveolar damage in the proliferative phase (P2) and in the organizing/fibrotic phase (P1 and P3) with fibroblastic proliferation and collagen fiber deposits (Figure 3D). All tissue samples had common morphological characteristics under electron microscopy. Around the capillaries, a large amount of cell debris was observed due to injury to type I and II pneumocytes with degeneration of the endothelium (Figure 3C). 

**FIGURE 4:**
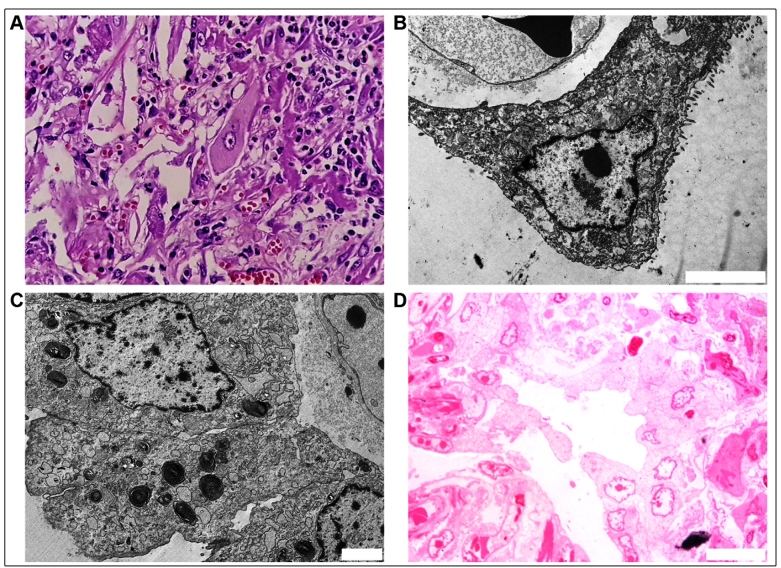
**(A)** Atypical pneumocytes with enlarged nuclei, and prominent binucleated nucleoli (Hematoxylin & eosin, 400x). **(B)** and **(C)** Electron microscopy of the lung showing hyperplastic type II pneumocytes displaying striking cytological atypia including cytomegaly, nucleomegaly, clearing of nuclear chromatin, and prominent nucleoli (B scale bar = 5 µm; C scale bar = 2 µm). **(D)** Semi-thin section of alveoli lined with type II pneumocytes in the proliferative stage occupying the alveolar space (AS) by optical microscopy (scale bar = 20 µm).

Ultrastructural studies of the inflammatory reaction during the proliferative stage revealed pronounced proliferation of type II pneumocytes (Figures 3 and 4) covered by hyaline membranes; this finding is associated with thickening of the septal walls and subsequent development of mural thickening. Furthermore, giant, multinucleated type II pneumocytes, large nucleoli, and dysplastic cells were also observed. The alveolar space, which was previously filled with exudates and type II pneumocytes, was replaced by proliferating fibroblasts with associated collagen and reticulin fiber deposition, as well as obliteration of alveolar spaces (Figure 4C).

**FIGURE 5:**
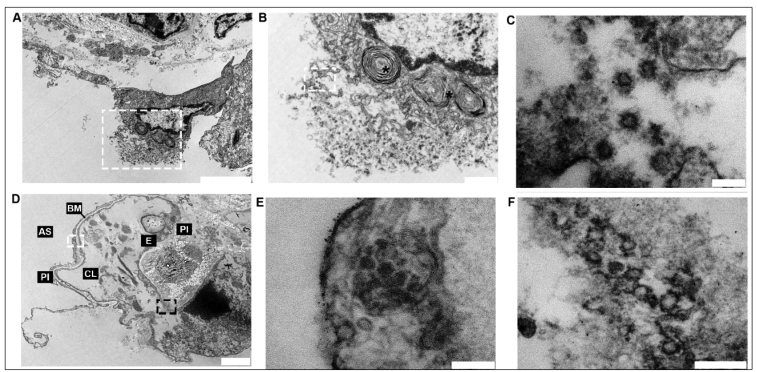
**(A)** Viral cytopathic effect in a type II pneumocyte, with viral particles inducing cytoplasmic membrane lysis (scale bar = 5µm). **(B)** Higher magnification of the dashed area in image A (*, lamellar bodies) (scale bar = 1µm). **(C)** Higher magnification of the dashed area in image B showing viral particles (scale bar = 0.1 µm). **(D)** Tissue degeneration observed in type I pneumocytes (PI), the basal membrane (BM), and endothelial cells (E) surrounding the capillary lumen (CL) (AS: alveolar space) (scale bar = 2µm). **(E)** Higher magnification of the white dashed area in image D showing viral particles in cytoplasmic vesicles in type I pneumocytes (scale bar = 0.1 µm). **(F)** Higher magnification of the black dashed area in image D showing viral particles in a damaged endothelial cell (scale bar = 0.2 µm).

Isolated and aggregated viral particles, each measuring 70 to 90 nm with peripheral spike projections and other characteristics consistent with SARS-CoV-2, were predominantly seen 1) in the cytoplasmic vesicles pneumocytes (Figure 5), similar to what has been previously described^6^ ^,^ ^7^; in the endothelial cytoplasm; or freely circulating in the alveolar space. Evidence of cellular damage was found in these pneumocytes and is characteristic of the cytopathic effect produced by the virus (Figure 5A and 5B). In addition, extensive degeneration, pneumocyte necrosis, and destruction of the capillary endothelium were also observed. (Figure 5D and 5E). 

## DISCUSSION

This study showed the ultrastructural changes that occur in the lungs of patients who die of COVID-19. We observed areas with intense and diffuse alveolar lung injury, as well as areas with evidence of an ongoing reparative process and parenchymal organization. Our findings regarding the exudative phase of DAD, which include desquamated pneumocytes and the presence of hyaline membranes, alveolar edema, fibrin deposits, and type II pneumocyte hyperplasia^8^, are similar to those described in previous literature. 

The initial changes seen in the exudative phase are characterized by alveolar and septal edema, congestion, and leakage of plasma into the intra-alveolar space; however, inflammatory infiltrates were scarce in the cases reported here. Lesions in the microcirculation also develop simultaneously, and we found exuberant reparative phenomena characterized by re-epithelialization of the alveolar epithelium mainly by type II pneumocytes. Electron microscopy demonstrated apoptotic changes in type II pneumocytes in patients with idiopathic pulmonary fibrosis/usual interstitial pneumonia (IPF/UIP). These changes could be the principal cause of several events that account for the histological, clinical, and functional alterations observed in these patients^9^, and apoptosis of these cells is implicated in the pathogenesis of lung injury^10^. 

An important finding associated with type II pneumocyte hyperplasia is thrombotic microangiopathy; this includes dilation of the capillary lumina, loss of basement membrane electron density, thrombi formation, and rupture of vessel walls. Platelet and fibrin aggregations were observed inside the lumen, while with capillary alveolar hemorrhage foci could be seen on the outside. Varga et al.^11^ found evidence of endothelial cell dysfunction in previous autopsies. Furthermore, they showed the presence of viral inclusion bodies in the renal peritubular space, as well as viral particles in the endothelial cells of the glomerular capillary loop. These results suggest that the presence of viral elements within endothelial cells and the accumulation of inflammatory cells facilitate the induction of endothelitis. However, we did not encounter this phenomenon in our patients, which is similar to other reported cases^8^ ^,^ ^12^. Ackermann^13^ observed the presence of virus-like particles within lung endothelial cells in a patient with COVID-19. This nonspecific cellular damage to capillary endothelial cells and the basement membrane leads to microvascular thrombosis with accompanying hemorrhage; these findings were also observed in our study. Therefore, we cannot wholly exclude the possibility of direct, virus-induced damage to the endothelium. 

This study used electron microscopy to propose a possible pathogenesis for SARS-CoV-2-induced lung injury. The proposed pathogenesis is based mainly on alveolar damage causing degeneration and necrosis of epithelial cells, appearance of the viral cytopathic effect in pneumocytes, and secondary inflammation. The presence of diffuse alveolar damage with the formation of intra-alveolar fibrin deposits, hyaline membranes, or loosely organizing connective tissue in the septal walls and alveolar spaces has been shown in other autopsy series^12^. The other characteristics observed in this study, such as cytomegaly without viral inclusions, giant cell formation, and desquamation of pneumocytes, have already been described in the first outbreak of SARS-associated coronavirus (SARS-CoV)^14^, as well as in other viral respiratory diseases such as swine-origin influenza type A^15^. As in the study on SARS-CoV^16^, the present study showed hyperplastic type II pneumocytes with similar cytological alterations, including cytomegaly, karyomegaly, clearing of nuclear chromatin, prominent nucleoli, and multinucleation. In our case, autopsies of the COVID-19 cases showed some similarity with those described for H1N1 in 2009^17^, and with H5N1 infection in humans^18^; however, the alveolar damage caused by COVID-19 appears to be much more aggressive. 

Similar to our findings, other studies on COVID-19 have described the formation of pulmonary interstitial fibrosis and microthrombosis^19^. Bradley et al.^12^ described the presence of coronavirus-like particles inside tracheal epithelial cells and in the extracellular space, or as a mix with luminal mucus. Extensive sloughing of pneumocytes into the alveolar spaces and, occasionally, the presence of viral particles, were observed within the vesicles of type I and II pneumocytes. The coronavirus is a member of the *Coronaviridae* family, which is formed by enveloped viruses with surface projections commonly found in intracytoplasmic vesicles^20^. However, careful and comparative examination of all electron microscopy structures of viral particles is necessary. Other cellular structures, such as microvesicular bodies, clathrin-coated vesicles, parts of the endosomal pathway^21^, or structures with cross-sections of the rough endoplasmic reticulum^20^, can be confused with viral structures. These pitfalls can occur especially when ultrastructural studies are performed in samples with numerous artifacts due to autolytic processes or inadequate fixation^22^.

This is the first report from Brazil to describe lung epithelial and vascular alterations using electron microscopy in Amazonian patients with COVID-19. We observed diffuse alveolar damage in the proliferative and fibrotic phases, which is probably due to the long period of hospitalization of these patients. Our ultrastructural findings suggest that the main mechanisms of cellular injury in the pulmonary parenchyma are related to the proliferation and lysis of pneumocytes secondary to the viral cytopathic effect that induces the subsequent formation of inflammatory lesions. The vascular effects of viral infection, such as disruption of the endothelial barrier, increased vascular permeability, formation of endothelial lesions, and thrombosis, also contribute to lung injury. On the other hand, patients who survived the acute phase developed scarring lesions with fibroblast proliferation and collagen fiber deposits. Further ultrastructural studies are needed to fully describe the pathogenesis of COVID-19 in different tissues. 
